# Evaluation of Three State-of-the-Art Classifiers for Recognition of Activities of Daily Living from Smart Home Ambient Data

**DOI:** 10.3390/s150511725

**Published:** 2012-05-21

**Authors:** Tobias Nef, Prabitha Urwyler, Marcel Büchler, Ioannis Tarnanas, Reto Stucki, Dario Cazzoli, René Müri, Urs Mosimann

**Affiliations:** 1 Gerontechnology and Rehabilitation Group, University of Bern, Bern 3010, Switzerland; E-Mails: prabitha.urwyler@artorg.unibe.ch (P.U.); marcel-buechler@bluewin.ch (M.B.); ioannis.tarnanas@artorg.unibe.ch (I.T.); reto.stucki@artorg.unibe.ch (R.S.); dario.cazzoli@artorg.unibe.ch (D.C.); rene.mueri@insel.ch (R.M.); urs.mosimann@gef.be.ch (U.M.); 2 ARTORG Center for Biomedical Engineering Research, University of Bern, Bern 3010, Switzerland; 3 Division of Cognitive and Restorative Neurology, Department of Neurology, University Hospital Inselspital, University of Bern, Bern 3010, Switzerland; 4 University Hospital of Old Age Psychiatry, University of Bern, Bern 3010, Switzerland

**Keywords:** healthcare technology, smart homes, smart cities, ambient assisted living, activities of daily living, data classification, data mining

## Abstract

Smart homes for the aging population have recently started attracting the attention of the research community. The “health state” of smart homes is comprised of many different levels; starting with the physical health of citizens, it also includes longer-term health norms and outcomes, as well as the arena of positive behavior changes. One of the problems of interest is to monitor the activities of daily living (ADL) of the elderly, aiming at their protection and well-being. For this purpose, we installed passive infrared (PIR) sensors to detect motion in a specific area inside a smart apartment and used them to collect a set of ADL. In a novel approach, we describe a technology that allows the ground truth collected in one smart home to train activity recognition systems for other smart homes. We asked the users to label all instances of all ADL only once and subsequently applied data mining techniques to cluster in-home sensor firings. Each cluster would therefore represent the instances of the same activity. Once the clusters were associated to their corresponding activities, our system was able to recognize future activities. To improve the activity recognition accuracy, our system preprocessed raw sensor data by identifying overlapping activities. To evaluate the recognition performance from a 200-day dataset, we implemented three different active learning classification algorithms and compared their performance: naive Bayesian (NB), support vector machine (SVM) and random forest (RF). Based on our results, the RF classifier recognized activities with an average specificity of 96.53%, a sensitivity of 68.49%, a precision of 74.41% and an F-measure of 71.33%, outperforming both the NB and SVM classifiers. Further clustering markedly improved the results of the RF classifier. An activity recognition system based on PIR sensors in conjunction with a clustering classification approach was able to detect ADL from datasets collected from different homes. Thus, our PIR-based smart home technology could improve care and provide valuable information to better understand the functioning of our societies, as well as to inform both individual and collective action in a smart city scenario.

## 1. Introduction

The prevalence of dementia in Western countries is steadily increasing, and improving the management of dementia symptoms and care has become a primary priority for socioeconomic reasons [1]. Usually, functional impairment is not clearly evident in prodromal dementia patients, and its measurement is therefore not feasible. However, in patients with later prodromal stages closer to overt dementia and in patients with mild Alzheimer’s disease (AD), subtle impairments of function are measurable. In these populations, the assessment of the activities of daily living (ADL) and of the instrumental activities of daily living (IADL) is useful to evaluate the impact of medicinal product-related improvements in everyday function [2]. Self-ratings might be applicable in milder disease stages, while in advanced disease stages, measurements largely rely on the reports of relatives or caregivers in close and regular contact with patients. Additionally, some methods of measurement exhibit both gender and culture biases. Several scales have been proposed to measure either basic ADL (or self-care) that relate to physical activities (such as toileting, mobility, dressing and bathing; [3]), or IADL (such as shopping, cooking, doing laundry, handling finances, using transportation, driving and making phone calls; [4]). Although IADLs are found to decline from the early and pre-diagnostic stages onward [2,5], the focus of research on common self-care or domestic activities disregards many other activities that may have gained more relevance in recent times (e.g., the use of technology). This results in low sensitivity to change of most of the assessment scales currently in use. In contrast, the ability to perform ADL deteriorates more rapidly during the later stages of dementia. Several studies have shown that ADL are an important predictor of quality of life [6,7]. Moreover, Giebel *et al.* [2], in a study with 122 dementia patients, found that the Katz Index of Independence in Activities of Daily living [4] correlates with the Quality of Life in Alzheimer’s disease rating scale [7].

In this context, smart home-specific measurement tools of ADL/IADL for early and advanced disease stages are needed, which add new dimensions to the existing assessment tools and allow a better evaluation of clinically meaningful changes. So far, impairments in four IADL items (handling medications, transportation, finances and telephone use) have been shown to be the most sensitive indicators of early stages of dementia (particularly when performance speed is taken into consideration). In contrast, basic ADL (such as toileting, dressing and bathing) are sensitive indicators of change in advanced disease stages [8].

Recognizing daily activities in smart home settings using in-home sensors is a well-researched problem [9,10,11,12]. In a recent review [12], Peetoom *et al.*, performed a meta-analysis of monitoring technologies that detect ADL or significant events (e.g., falls of elderly people in-home) and identified five main types of monitoring technologies with the goal of prolonging independent living. The identified technologies were PIR motion sensors, body-worn sensors, pressure sensors, video monitoring and sound recognition, most frequently combined in a multi-sensor approach. The authors concluded that, although monitoring technology is a promising field, the technologies themselves have to be brought to the next level and integrated inside a smart city environment. Longitudinal studies are thus needed to evaluate their (cost-) effectiveness and to demonstrate the potential to prolong independent living of elderly persons in a pervasive smart city environment.

Following the aforementioned meta-analysis, we will outline a few existing smart home solutions that use simple sensors, such as multi-sensor approaches. They were used to detect emerging patterns of frailty by analyzing movements of the resident from one room to another (*i.e.*, motion sensors in the doorway) and changes in the state of objects and devices (*i.e.*, contact sensors). For instance, Kasteren *et al.* [13] used temporal probabilistic models (naive Bayes, hidden Markov models and conditional random fields) to recognize activities from sensor readings, dividing time series data into time slices of constant length and labeling the activity for each slice. However, the authors did not consider the duration of such activities. Moreover, defining a constant length time slice for all activities may not be practical. In a later paper by the same authors [14], hidden semi-Markov models were used, which consider the duration of a given activity in order to improve the accuracy of their recognition.

Recent works [15,16,17] have focused on recognizing concurrent and interleaved daily activities. For instance, Zhang *et al.* [17] used activity durations for activity recognition. They proposed an algorithm to learn different time slice durations for different activities from the training data. They then used these durations for building models of different activities. A common problem associated with the works discussed so far is that they require accurate labeling of activities during training, performed either by the resident or by the experimenter, who has to manually annotate the data after viewing. This kind of data analysis may be difficult to obtain for long time periods. In addition, a smart home environment might provide the data necessary for an automatic annotation.

Within that context, Kasteren *et al.* [18] presented a technique allowing the use of the ground truth collected in one house to train activity recognition systems for other houses. However, the details of the activities may vary significantly from person to person and from home to home, in which case this technique may not perform well. Alternatively, Zhang *et al.* [19] presented an unsupervised technique that clusters the sensor firings using mixture models and that entails a self-adaptive neural network to summarize the timing of sensor firings for each activity. Similarly, Ordonez *et al.* [11] presented an unsupervised approach for activity recognition based on activity model extraction from sets of text, such as from the web, without any human labeling. This is done by first mining a set of object terms for each activity class from the web and then mining contrast patterns among object terms based on emerging patterns. These patterns are then used to automatically produce labeled segmentations of activity data. However, such background knowledge may not be available in every smart home. Furthermore, none of the unsupervised solutions mentioned so far address the problem of overlapping activities, which may degrade labeling performance.

Video-monitoring systems are an alternative to the aforementioned fully sensor-based equipped smart home approaches. In fact, video-monitoring systems can replace ambient sensing or be used in parallel to reduce the number of sensors necessary to describe the overall activity of a person. Some already developed applications vary from fall detection to ADL detection in constrained environments [20,21,22,23,24,25]. For instance, Maki *et al.* [23] presented an ontology-based approach, which has been shown to accurately model the context of human status (e.g., body posture) and the environment context using semantic information about the scene. These models used information provided by a set of cameras for person detection and by accelerometer devices attached to objects of daily living for environment event triggering (e.g., TV remote or cabinet use). A rule-based reasoning engine was then used for processing and combining both models types at the activity detection level. Moreover, the ontology tried to solve the semantic gap among the human activities and the sensors’ raw signals. In another approach [26], a fuzzy logic scheme was proposed to cope with multiple sensor activity analysis fusion in a smart home. Audio, infrared sensors, a wearable device (such as electrocardiography, ECG) and body posture were combined to infer ADL events.

However, although video surveillance of daily activities can support the analysis of medium-to-long-term patterns, it represents a highly intrusive approach. Low-cost, low-sensor ICT supported clinical protocols have thus been recently proposed, in order to analyze a person’s performance in specific activities (such as ADL) and to highlight potential emerging symptoms of specific diseases. For instance, wearable devices have been used to assess older peoples’ motor function performance, to identify disturbances in gait patterns that could be associated with disease progression, to personalize patient care, to assess the independent living associated risks and to decide on institutionalization. Yet, although a multi-sensor approach enriches the quantity of data on the person’s daily routine, multiple sources of the readings also increase the complexity of the data analysis process. It is therefore necessary to choose an easy, low-cost, low-level sensor system, which is able to detect the activity of interest, disregarding extant data storage issues.

In the present paper, according to our novel smart home approach to identify different ADL by using passive infrared (PIR) sensors, room temperature and light data [27], we evaluate the performance of three machine learning algorithms with respect to their sensitivity and specificity in correctly recognizing the activities performed by older people living alone. We hypothesized that the room-based clustering groups would represent an ADL. We further explored if these clustering groups can be automatically detected from the raw sensor firings, which would enable users to label each group as an activity just once, allowing all corresponding instances of this group to be automatically labeled. The main contributions of our system for a smart city scenario are: (1) a novel framework for training activity recognition systems that includes segmentation, mining and clustering of low-level smart building sensor events; and (2) an activity recognition system based on active learning classification that automatically recognizes new room-level occupancy episodes as members of one of the clusters (*i.e.*, bath activities), constructed during training.

## 2. Methods

A custom-made wireless sensor, smart building network was developed for the present study. The requirements were: (1) being able to recognize eight different activities of daily living; (2) being quick and easy to install; (3) not requiring any ambient assisted living (AAL) infrastructure; (4) not using body-mounted sensors; (5) being inexpensive; and (6) not requiring any active intervention of the resident.

### 2.1. Data Acquisition: Measurement Setup

The sensor network consists of ten wireless sensor boxes, one wireless protocol device and a laptop to store the captured data [28]. Each of the sensor boxes (*l* × *w* × *h* = 15 mm × 30 mm × 60 mm, weight = 80 g) measures ambient motion, temperature (°C) (Dallas DS18B20, Maxim Integrated, Munich, Germany), luminescence (l×) (AMS302, Panasonic Corporation, Holzkirchen, Germany) and humidity (g/m^3^) (SHT21P, SENSIRION, Staefa ZH, Switzerland), as well as the acceleration (m/s^2^) (ADXL345, Analog Devices, Munich, Germany) of the sensor box itself, at a rate of 0.2 Hz. The ambient motion is measured using a passive infrared radiation (V) (EKMB1101111, Panasonic Corporation, Holzkirchen, Germany) sensor, and the analysis presented herein is mostly based on the PIR data. A receiver unit is connected to a commercially available laptop, in order to collect the data packages sent from all ten sensor boxes. The laptop served as a central computing unit to process the environmental data and served as a data storage unit for further analysis.

Ten healthy subjects were recruited to monitor activities for 20 days using the sensor system. All subjects signed an informed consent, and the local ethics committee approved the data collection. The system was setup in the home of the subjects by placing the sensor boxes in the rooms. Each sensor box was placed in such a way that it could oversee the whole room. In flats, where the dining table is placed inside the living room, two sensors were set up, one of them targeting the table and the other observing the sofa. Additional sensor boxes were placed in the kitchen (on the fridge door) and in the bathroom (on the flush handle).

For validation, a wireless protocol device, built into a housing with a wearable belt clip, was provided to the subjects. The protocol device was fitted with switches, each corresponding to an allotted ADL. All subjects were instructed to flip the switches corresponding to the performed activities. This is how the logbook of the performed activities was obtained.

### 2.2. Data Preprocessing

In the first step, the sensor node number was associated with the corresponding room using information protocoled during the system setup. For comparability between datasets, similar rooms were labeled with the same code. After establishing this basic nomenclature, the data were rearranged into a format more suitable for the clustering and classification. In the reformatted file, for each room, three feature vectors were created: temperature, luminescence and PIR data. Apart from the aforementioned columns, an additional humidity feature was created for the bathroom, while additionally, four acceleration features were created for the fridge door sensor. As an additional feature, the weekday was also introduced. The data were reordered against time. A 5-s grid was established, and each measurement was assigned to the nearest time point. After transformation, the data were ordered in a tabular fashion with one row for every 5 s and the sensor data of all measured rooms into the corresponding feature columns. The information from the logbook was transformed in a similar way.

Most ADL have spatial regularity. For example, people cook in the kitchen and sleep in the bedroom. Therefore, the first step of our training algorithm was to segment consecutive sensor firings based on in which room the sensors were. We approached this problem with the aforementioned insight that different daily activities are performed in different rooms. Each activity triggers a different set of sensors to fire and is often performed during similar times of the day. Therefore, we divided the sensor firings into room-level occupancy episodes, segmenting and clustering a time series of sensory data into intervals (unsupervised part). Then, for each room, we classified these time intervals using the logbook (supervised part).

### 2.3. Data Analysis

**Figure 1:**
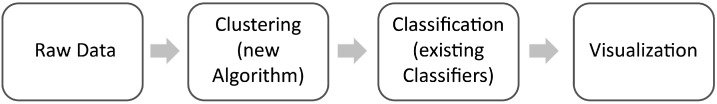
Starting with the (reformatted) raw data, a clustering further preprocessed the data before the actual classification was performed. Finally, the computed result was displayed.

The entire data mining and analysis process was based on existing classification algorithms. In order to obtain optimal results, data clustering was performed prior to data classification, using a clustering algorithm specifically tailored for our data. In order to choose and develop the ideal components for a comparable metric of performance, we used the sensitivity and specificity given by a leave-one-out cross-validation. Figure 1 shows an overview of the whole process.

#### 2.3.1. Classification

The goal of classification was to assign measured ambient sensor values to a given set of classes.

In our case, these classes were the different ADL. We trained the classifier using a set of exemplary data with corresponding class labels from which we named the training set. All measurements with corresponding logbook entries were fed into the classifier, which then trained it to give the best predictions. Three well-established classifiers (naive Bayes, support vector machine and random forest), all of which use the supervised training approach, were used for ADL classification. They differ fundamentally in their approach on how to classify data. The choice of these three classifiers was based on common practice, for the NB and SV, and on the novelty and resistance to over-fitting performance, shown by many data mining and machine learning researchers, for RF [29].

##### Naïve Bayes Classifier

This classifier is based on Bayes’ theorem [30], which assumes that the features are independent. Bayes’ conditional probability model is then combined with a decision rule, which picks the most probable hypothesis. This is done by maximizing the posterior probability, thereby assigning a class label to a given input vector.

##### Support Vector Machine Classifier

SVMs [31] are another well-known and established way to classify data in a non-probabilistic manner. Initially, this classifier required linear separability of the classes. However, nowadays, many different kernel functions (linear, polynomial, Gaussian, radial basis function) are used with SVMs to classify all sorts of data with any sort of hyper-planes. SVMs can be considered as one of the fundamental non-probabilistic classifiers and are widely used [29].

##### Random Forest Classifier

The RF classifier [32] belongs to one of the newest algorithms and is a non-probabilistic decision tree-based classifier. The algorithm generates a number of decision trees. For each tree, only a random subset of the available data is considered. Additionally at each node, only a random subset of all features is used for the split. No pruning is performed on the final trees. To classify new data, they are fed into each tree, so that a majority vote over all trees decides which class label is assigned. The advantage of the RF classifier is its resistance to over-fitting, which guarantees generalization to new data.

**Figure 2:**
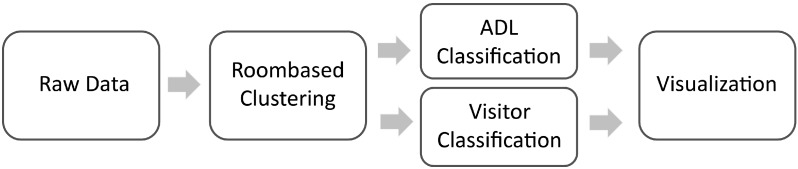
Additionally to the activities of daily living (ADL) classifier, a parallel visitor classifier was used. The results of the two classifiers were then merged.

In addition to the ADL classification, it was also necessary to determine at which times the subject was at home and when there was a visitor present. With data from the PIR motion sensors, periods without any activity in the flat were detected. As “having a visitor” cannot be defined as an ADL and the reliable detection of the frequency of visitors in the flat can be difficult, visitor detection was performed using a second classifier solely for this purpose. The visitor classification was thus completely independent from the ADL classification. Figure 2 illustrates the modified algorithmic process. The RF classification was used to recognize visitors.

#### 2.3.2. Clustering

Since our sampling rate was 0.2 Hz, our measured data were clearly oversampled, because ADL usually last much longer [3] than fractions of s. We thus grouped together similar data points. By grouping together similar data points, we were not only able to remove outliers, but also to compress the amount of information, decreasing therefore the classification duration. Moreover, the additional data-clustering step allowed specifically focusing on given features of the data and opened the possibility to introduce additional constraints.

**Figure 3:**
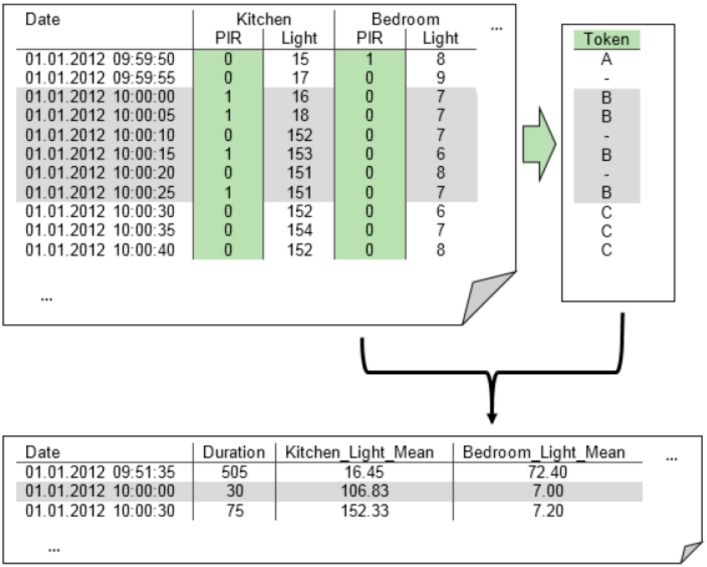
A token was calculated based on all passive infrared (PIR) values. Whenever the token changed (inactive states of all motion sensors were neglected), a change point was set. Periods between two change points were then compressed.

We developed our clustering algorithm tailored to our measurement data and non-intrusive sensor network using MATLAB. The initial clustering was solely based on the motion sensor values. A unique token for each PIR constellation was computed based on the PIR values of all rooms. Based on this token sequence, a clustering was performed. Whenever the token changed (meaning that other movement sensors were active), a change point was set, and changes to motionless periods were neglected. The time periods between two change points were compressed into one data row by computing the mean and variance for each feature (column) of the data, as depicted in Figure 3. Finally, the computed token code was added in a new column, as well as the token code of the previous and next time period. This procedure added some information about the continuity to each data point and improved classification by embedding each time period into a context.

Based on various validations of the initial clustering, a more sophisticated room-based clustering was implemented. The following assumptions built the foundation for the clustering algorithm:

(1) The room in which the subject was located had a major influence on the possible ADL.
(2) An ADL was usually quite a long event (minutes up to hours), with the exception of toileting. Therefore, short “disturbances” could be neglected for most rooms.
(3) When differentiating between a person leaving the house or performing an ADL without measurable movement, the edges of the adjacent activity periods were important, even if those activities were very short.

In addition, during an activity period, the subject might have changed the performed ADL. To identify the occasions in which a subject moved between rooms, different averaging filters were used on the PIR data. Additionally, different weights were applied for certain rooms and/or room-filter combinations. For instance, short-term movements in the bathroom were weighted high, whilst movements in other rooms were more dependent on long-term activity. Resting on this filtering, several change points were determined. Moreover, during an activity period, ADL were expected to be quite long. However, useful information about the no activity period might be found at the edges of the activity periods, especially regarding whether the subject left the flat completely or not. Since the period of this information might be quite short, we filtered the edges of such activity periods differently, in order to retain short information peaks at their edges.

**Figure 4:**
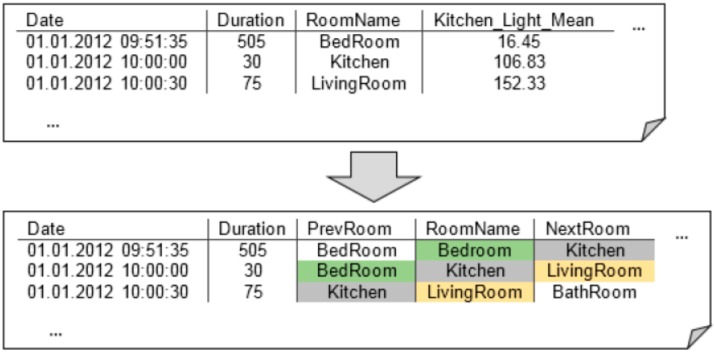
To provide the classifier with contextual information about overlapping time periods, additional feature columns were introduced.

Finally, the two lists of the activity/no activity and room-based change points were merged, and the time periods between two change points were compressed into a single data point (row) by calculating mean, variance and other key figures for each feature (column). For each activity period, the most likely spatial location of the subject in the flat was estimated, based on the PIR values. In order to provide the classifier with more contextual information about the ongoing activity, time periods were further characterized by saving the corresponding room name, duration and activity degree. An example of this step is illustrated in Figure 4.

### 2.4. Classification Performance

To evaluate the activity recognition performance of the NB, SVM and RF classifiers, we performed leave-one-out [33] cross-validation on the ten datasets. During each step of cross-validation, we trained our system with the nine datasets, which generated a set of clusters. We then used the trained system on the remaining dataset (tenth) to label its room occupancy episodes as instances of the clusters. The activity log from the wireless protocol device served as the ground truth for comparison with the classifier’s output. We thus obtained the activity labels and compared them with the ground truth to calculate the time slice error. Metrics, such as sensitivity/recall, specificity, precision and the F-measure [34,35], were used to evaluate the performance of the classification. The performance metrics (sensitivity, specificity, precision, F-measure) were calculated by cross-validating the output of the classifiers with the ground truth.

### 2.5. Software/Tools

The algorithms were developed using MATLAB (MATLAB R2012 b, The MathWorks, Inc., Natick, MA, USA), whilst KNIME (KNIME 2.9.2, windows 32 bit version) [36] with the Weka plugin (Version 3.7) [37] was used for the data analysis. MATLAB was used for data conversion, processing and clustering. The transformed data was then loaded into KNIME, where the classification was performed using the aforementioned classifier of the Weka toolbox.

## 3. Results

**Figure 5:**
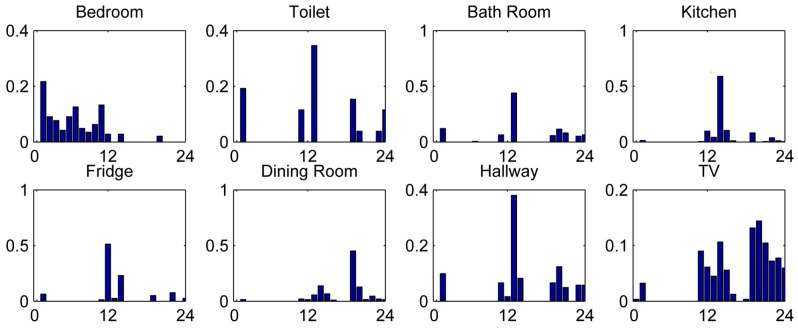
Distribution of PIR recordings during 24 h of measurements for one volunteer. The x-axis shows the time of the day and the y-axis the normalized number of PIR recordings.

The dataset for this study was collected in 10 homes of 10 healthy volunteers (four men, six women) of various ages (min = 28; max = 79; mean = 48.8) during 20 days each. This led to a cumulative observation period of 200 days. Within the 200 observed days, participants logged 343 ADL. A typical dataset is presented in Figure 5. The figure shows a distribution of the PIR recordings in different rooms, separated by the time of day.

### 3.1. Classification Performance

Table 1 shows the performance metrics of the NB, SVM and RF classifiers. The RF classifier performed better than the NB classifier and SVM classifier. The NB classifier achieved the highest specificity for grooming (98.61%), seated activity (99.42%) and watching TV (97.24%), while performing worse on the rest of the ADL. In contrast, seated activity (1.35%) using the NB classifier achieved the lowest F-measure, and grooming (91.93%) achieved the highest F-measure using the RF classifier.

The performance of the RF classifier on visitor detection using all data (all rooms) yielded a sensitivity of 10.90%, a specificity of 99.15%, a precision of 43.85% and an F-measure of 17.46%. These metric values increased to 21.08%, 99.88%, 92.11% and 34.31%, respectively, while considering data only in certain rooms, such as the bathroom, TV room and living room.

**Table 1:** Performance (sensitivity (recall), specificity, precision, F-measure) comparison of naive Bayes (NB), support vector machine (SVM) and random forest (RF) classifications in a leave-one-out cross-validation on token clustered data.

	Sensitivity (Recall)			Specificity			Precision			F-Measure		
	NB	SVM	RF	NB	SVM	RF	NB	SVM	RF	NB	SVM	RF
Cooking	25.02	56.86	58.85	92.31	89.72	97.50	18.70	28.11	62.49	21.41	37.62	60.62
Eating	5.60	17.96	42.82	94.90	96.52	99.58	4.18	17.00	80.32	4.79	17.47	55.86
Get ready for bed	78.23	38.47	69.21	78.58	95.18	98.80	34.87	53.94	89.41	48.24	44.91	78.02
Grooming	27.99	62.19	95.33	98.61	95.69	94.86	89.54	86.02	88.76	42.64	72.19	91.93
Seated activity	0.71	23.21	34.80	99.42	91.63	97.87	12.96	25.06	66.28	1.35	24.10	45.63
Sleeping	20.93	56.41	79.89	96.47	93.78	98.64	27.58	36.81	79.03	23.79	44.56	79.45
Toileting	49.89	37.73	82.18	61.58	83.14	92.67	12.58	19.88	55.42	20.09	26.04	66.19
Watching TV	5.34	40.59	84.84	97.24	93.04	92.33	32.78	59.54	73.62	9.19	48.27	78.83
Mean	26.72	41.68	68.49	89.89	92.34	96.53	29.15	40.79	74.41	27.88	41.23	71.33

### 3.2. Clustering Performance

After excluding short activities (less than 20 s), the cross-validation performance with the RF classifier improved to 73.78% sensitivity and 96.85% specificity. With the final room-based clustering, a sensitivity of 79.51% and a specificity of 98.90% were obtained with the RF classifier.

## 4. Discussion

Our wireless sensor network achieved an accurate labeling of all ADL activities using the RF classifier with an average specificity of 96.53%, sensitivity of 68.49%, precision of 74.41% and F-measure of 71.33%. This result supports the ease of use of the system in practical deployments, since the classifier did not need the ground truth for all instances of all ADL during training. The performance of the system was good enough using common practice (NB, SVM), but much better using state-of-the-art classifiers (RF) and in line with other studies [38].

We compared the classifier performance by dividing raw sensor data into fixed-length time slots and classified each slot based on whether the sensors fired within that slot. We set the time slot length to 60 s, because this resulted in the highest accuracy for the system. For some activities (*i.e.*, “seated activity”, “eating”, “cooking”), some instances took place at unusual times (e.g., the resident ate at midnight or went to sleep at 3 AM). Nevertheless, such instances occurred rarely in the entire dataset.

The practical problem that still needs to be addressed is represented by overlapping activities. For example, people may leave the kitchen in the middle of cooking to do something else, such as eating, and return to finish cooking. Alternatively, people may cook and have a drink at the same time, while in the kitchen. Our system considered the instances above as normal, which results in an overall lower sensitivity for these activities. However, NB, SVM and RF performed equally well for these activities. In fact, these activities only depend on a single sensor, and the algorithms made decisions based on the firings of that particular sensor, ignoring temporal characteristics. NB performed much worse than the other classifiers, because it classifies each time slot independently, without considering the previous activity or the durations of the current or previous activity.

Only low sensitivity was achieved for visitor detection due to the fact that the current setup is only able to detect visitors when the two persons stay in different rooms. With the given sensor boxes, it is not possible to distinguish if one person or many persons are in the room. It would be favorable to detect visitors whenever they enter or leave the flat. Sensors directed at the dressing area of the hallway or even at the door, such that door openings can be detected, may improve visitor detection.

One limitation of our system is that it was evaluated only on the data we collected. Our techniques can be generalized and be applied to any dataset from a single-person, multiple-room home. However, this will need some manual work in data preparation with respect to the number of sensors, sampling frequency and formatting. In particular, we plan to extend our system for multi-person homes in the future, by associating each sensor firing with the corresponding user who triggers it. If such data association is shown not to be possible, we plan to use the variation in temporal characteristics of how different users perform an activity.

Another limitation is that the segmentation step assumes that there are multiple rooms in the house, and thus, segmentation would not work with only one room. In such scenarios, we would need to design new techniques for the segmentation. Along the same lines, although some results show many ADL instances as irregular (e.g., seated activity for less than 60 s), the user might have started performing that activity in a different way after the training. Therefore, ways to incorporate new trends in behavior should be adjusted by periodically re-training the system.

The data presented in our study has been recorded in an opportunistic way, and it is most likely that our classification was based on an unbalanced dataset. Learning from unbalanced datasets can deteriorate classifier performance, and different performance metrics have been evaluated to take into account the class imbalance [34,35]. Although RF classification achieved the best results on our data, balanced random forest [39] and weighted random forest [39] may further improve the classification results.

In spite of being an automated approach relying on PIR, temperature and light data only, our system performs as good as some of the state-of-the-art supervised activity recognition systems [20,22,24,25,26]. None of these supervised techniques take the time of the day into account during activity recognition. If they were implemented in a way that the time of day were also to be considered, then their performance could potentially improve, and in that case, our system might perform slightly better. However, our system has the benefit of using classifiers, such as RF, and a significantly lower number of user-labeled activity instances in the training set.

## 5. Conclusions/Outlook

Opportunistic sensing of a variety of behaviors in smart city settings can be achieved via activity recognition (AR) platforms now provided by remote wireless sensors. The recorded multivariate sensor streams undergo analysis in order to infer the activities that were performed by the subject.

The preliminary results of our novel smart home methodology proposed here show that the adoption of a PIR sensor approach with automatic RF classifiers can improve event detection performance of a smart home AR system. Despite these promising preliminary results, further work, with a larger scaled dataset and collected with multiple and more sensitive PIR nodes, is required to increase the significance of the obtained results. Future work will focus on a broader validation, which is planned to evaluate the reproducibility of the results in a larger number of patients. The proposed PIR sensor approach could also support the calculation of the likelihood of events based on multiple PIR sensors readings, and this option will be considered in the next evaluation.

The long-term goal of the proposed approach is to support caregivers and clinicians in the identification of emerging symptoms of cognitive decline or possible diagnoses in a quantitative and objective way inside a smart city scenario. To better understand and improve the “health” functioning of our societies, the “health state” of a smart building is an important contributor. It includes the physical health of citizens, longer-term health norms and outcomes, as well as the arena of positive behavior changes.
